# A systematic review and exploration of clinical application of liver depression syndrome in breast cancer

**DOI:** 10.3389/fonc.2025.1614903

**Published:** 2025-08-11

**Authors:** Peng Wu, Siying Mao, Qian Zuo, Qianqian Guo, Qianjun Chen

**Affiliations:** 1 State Key Laboratory of Traditional Chinese Medicine Syndrome/Breast Disease Specialist Hospital, The Second Affiliated Hospital of Guangzhou University of Chinese Medicine, Guangdong Provincial Hospital of Chinese Medicine, Guangdong Provincial Academy of Chinese Medical Sciences, Guangzhou, Guangdong, China; 2 The Second Clinical College of Guangzhou University of Chinese Medicine, Guangzhou, Guangdong, China; 3 Guangdong Provincial Hospital of Chinese Medicine, The Second Affiliated Hospital of Guangzhou University of Chinese Medicine, Guangzhou, Guangdong, China; 4 Department of Breast, Guangdong Provincial Hospital of Chinese Medicine, Guangzhou, Guangdong, China; 5 Guangdong Provincial Key Laboratory of Clinical Research on Traditional Chinese Medicine Syndrome, Guangdong Provincial Hospital of Chinese Medicine, Guangzhou, Guangdong, China; 6 Postdoctoral Research Center, Guangdong Provincial Hospital of Chinese Medicine, Guangzhou, Guangdong, China; 7 Chinese Medicine Guangdong Laboratory, Guangzhou, Guangdong, China

**Keywords:** breast cancer, liver depression syndrome, systematic review, Chinese medicine syndrome, clinical application

## Abstract

Breast cancer remains one of the most prevalent malignancies among women. Traditional Chinese Medicine (TCM) identifies Liver depression syndrome as a core clinical pattern in breast cancer; however, the absence of systematic reviews addressing this specific syndrome has constrained the development and application of TCM interventions. This systematic review searched seven Chinese and English databases, and was supplemented by published books and clinical guidelines, to identify literature documenting TCM diagnostic indicators of Liver depression syndrome in breast cancer. The collated data formed the TCM item for Liver depression syndrome in breast cancer, and descriptive statistics were applied to analyze symptom patterns. This study revealed 104 distinct clinical manifestations and 56 tongue-pulse indicators. The predominant syndrome subtypes included syndrome of *qi* stagnation due to Liver depression (30.5%), syndrome of phlegm congealing due to Liver depression (24.7%), syndrome of Liver *qi* depression (19.7%), and syndrome of Liver depression and Spleen deficiency (15.6%). Key clinical manifestations comprised distending pain in the chest and sub-costal region (68%), depression (67%), breast mass (64%), anger (52%), breast distending pain (44%), and oppression in chest (44%). The most prevalent tongue manifestation was thin white fur, while the most common pulse pattern was the stringy pulse. The current findings highlight diagnostic inconsistencies in the definition of Liver depression syndrome in breast cancer, posing challenges to clinical research and the elucidation of syndrome mechanisms. Future investigations should prioritize the standardization and objective characterization of diagnostic criteria to enhance the accuracy of syndrome recognition and facilitate scientific validation.

**Systematic review registration:**

https://www.crd.york.ac.uk/prospero, identifier CRD42024546784.

## Introduction

Breast cancer constitutes a significant contributor to cancer-related mortality among women (1), and data from China show that its incidence ranks second among cancers (2). The pathogenesis of breast cancer involves multiple mechanisms, among which emotional factors play a key role in the occurrence and progression of this disease (3). Previous studies, including systematic reviews (4), multicenter clinical studies (5), and Delphi study (6), have consistently identified Liver depression syndrome as the main clinical pattern of breast cancer. Liver depression syndrome is a general syndrome term which contains a series of syndrome subtypes centered on constrained Liver *qi*. Based on the common etiology, different syndrome subtypes can be divided at different stages of pathology (7).

In TCM theory, it is believed that the core pathogenesis of Liver depression syndrome is attributed to chronic emotional constraint and psychosocial stressors. Characteristic clinical manifestations encompass depression, sighing propensity, and irritability (8). In breast cancer, multiple etiological factors beyond emotional dysregulation can precipitate Liver depression. For example, breast cancer patients can suffer chronic emotional depression due to gender role stress (9). Meanwhile, modern lifestyles including lack of sleep (10), sedentary behavior (11) and high-fat diet (12) can also disrupt Liver function through multiple mechanisms. Thus, under the cumulative effect of these factors, persistent stagnation of Liver *qi* gradually disrupts the inter-organ coordination of the Liver, Spleen and Kidney, as well as the homeostasis of *qi* and blood circulation (13). This pathological state initiates a cascade of pathophysiological processes, such as *qi* stagnation, blood stasis, phlegm accumulation and toxin accumulation. These abnormalities accumulate in the mammary tissue, ultimately contributing to the oncogenesis of breast cancer.

While diagnostic criteria exist for subtype patterns like the Liver depression with *qi* stagnation and Liver depression with phlegm coagulation (7), standardized diagnostic parameters specific to breast cancer-associated Liver depression syndrome remain undefined. The standardization of syndrome diagnosis holds significant implications for TCM clinical practice and research. Establishing unified diagnostic criteria for Liver depression syndrome would not only enhance the precision of syndrome differentiation in breast cancer management but also facilitate the standardized application of TCM therapeutic approaches in breast cancer prevention and treatment. Moreover, such standardization would provide essential support for developing integrated TCM and Western medicine diagnostic frameworks and promote the international dissemination of research outcomes. Building on this, the current study systematically consolidates literature evidence and performs evidence-based medical evaluations to establish a scientific framework for developing standardized diagnostic criteria for Liver depression syndrome in the context of breast cancer.

## Methods

The methods employed to identify relevant research articles were guided by the protocols outlined in the Cochrane Handbook for Systematic Reviews of Interventions. Additionally, this study was meticulously reported in strict adherence to the PRISMA guidelines. This systematic review was prospectively registered on PROSPERO (Registration ID: CRD42024546784).

### Literature search strategy

Searches were conducted in the following seven electronic databases: China National Knowledge Infrastructure (CNKI), Chongqing VIP Information database (CQVIP), Wanfang Database, Chinese Biomedical Literature Service System (SinoMed), PubMed, Cochrane Library, and EMBASE. The search scope has covered all records from the inception to January 2024. Using “Liver Depression” as the core term, the search strategy targeted 18 clinically defined subtypes of Liver depression syndrome, all of the subtypes are manifestations of different pathophysiologic conditions caused by the etiology of Liver depression. Additionally, supplementary print literature sources, including textbooks, monographs, clinical guidelines, and consensus statements, were systematically reviewed to collect information from the four diagnostic methods (including inspection, auscultation, inquiry and palpation). The complete search terms and protocols are detailed in **Supplementary File 1** (Chinese/English Search Terms).

### Study eligibility criteria

Inclusion criteria were: (1) Pathologically confirmed breast cancer diagnosis; (2) Records of TCM diagnosis for breast cancer (*Ru Yan* or breast cancer); (3) TCM syndrome differentiation confirmed as Liver depression syndrome; (4) Inclusion of comprehensive symptom descriptions and information on Chinese herbal interventions; (5) Literature addressing the etiology, pathogenesis, or clinical manifestations of Liver depression syndrome in breast cancer, including clinical observations and expert experience reports.

Exclusion criteria were: (1) Cell or animal experimental studies; (2) Duplicate publications (only one version retained); (3) Secondary analyses, including reviews and meta-analyses; (4) Clinical studies focusing on breast cancer comorbidities (e.g., postoperative lymphedema); (5) Studies involving diagnostic criteria for complex syndromes beyond Liver depression syndrome. In this study, no unpublished or original data were included.

### Data extraction

Researchers systematically extracted the following variables: author information, publication year, study type, geographic region, publishing source (journal/publisher), TCM diagnostic patterns of Liver depression syndrome, signs and symptoms, pathological classification, and cancer staging. For textbook sources, entries from the same series with different editors-in-chief were consolidated as a single entry, while multiple editions by the same editors-in-chief retained only the content from the most recent edition to ensure timelines and avoid duplication.

Symptom standardization primarily referenced *Clinical Terminology of Traditional Chinese Medicine Diagnosis and Treatment–Part 2: Clinical Patterns (GB/T 16751.2-2021) (*7), *Standardized Glossary of TCM Clinical Symptoms (*14), and the *Diagnostic Differentiation of TCM Symptoms (2nd Edition) (*15). This standardization process provided the basis for constructing a TCM item pool for Liver depression syndrome in breast cancer, ensuring terminological precision in the systematic review.

### Data analysis methodology

Literature quality assessment adhered to the hierarchical evidence grading system for TCM studies (16), which categorized sources as Level I (large-scale randomized investigations with definitive outcomes and minimal type I/II error risks), Level II (small-sample randomized studies with indeterminate results and elevated error probabilities), Level III (non-randomized concurrent controlled trials and historical expert consensus), Level IV (non-randomized historical controls and contemporary consensus statements), and Level V (case reports, uncontrolled observations, and expert opinions). Descriptive statistical analysis of TCM signs and symptoms associated with Liver depression syndrome in breast cancer was performed using SPSS 26.0, with frequency distributions calculated for all documented clinical symptoms.

## Results

**Figure 1:**
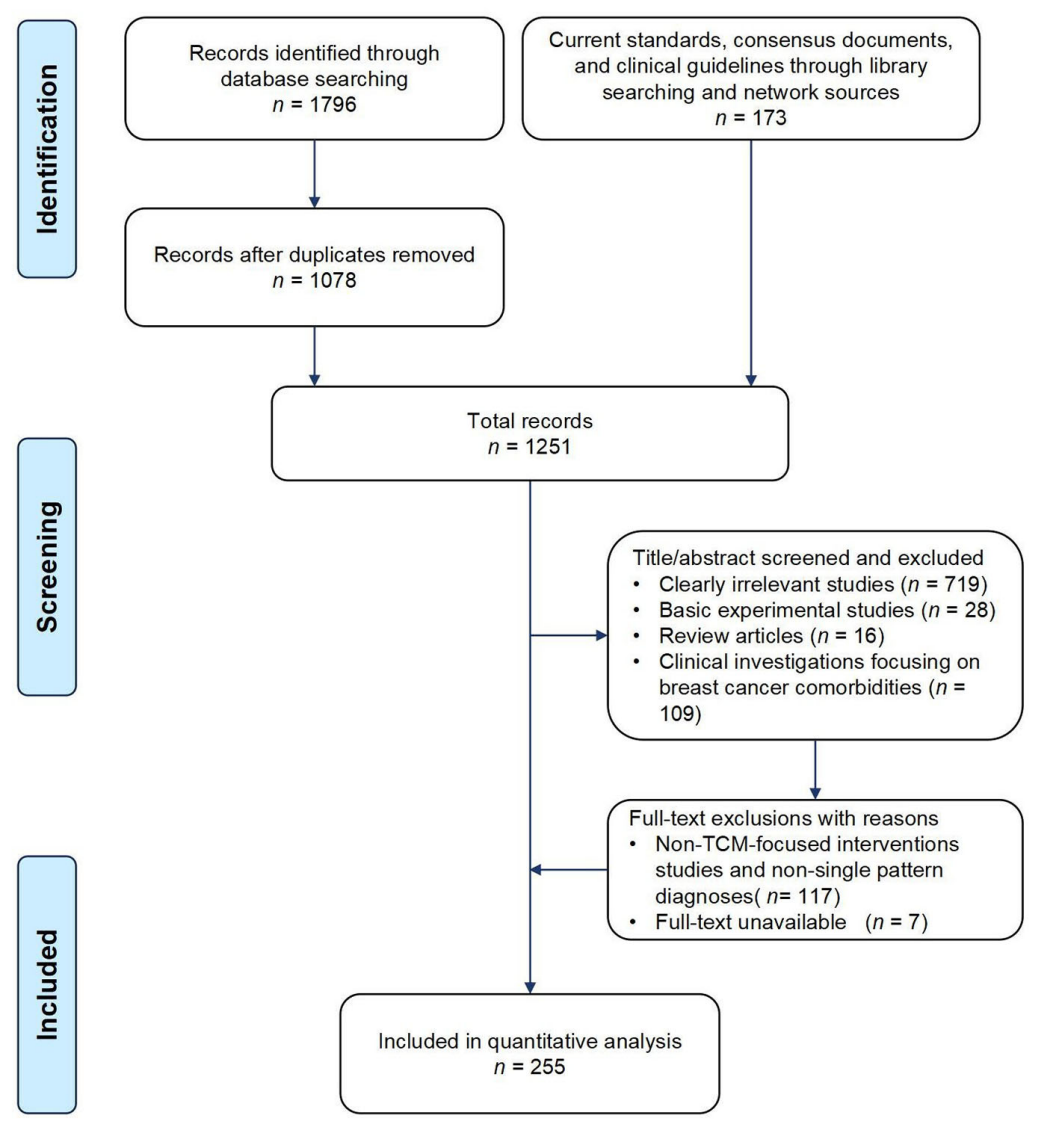
Flowchart of study selection of the published literature.

A total of 255 eligible literatures were screened, including eight expert consensus documents, 130 textbooks/monographs, 35 medical record files of renowned physicians, and 82 contemporary research articles. It was worth noting that due to the reporting of multiple subtypes of Liver depression syndrome in some literatures, thus, a total of 298 clinical entries were ultimately analyzed. The literature screening was shown in **Figure 1**.

### Characteristics of included studies

Among the 82 included studies, 46 were clinical experience summaries and 36 were clinical observations. Level V literature accounted for 56.1% (46/82), Level I literature accounted for 30.5% (25/82), and Level II literature accounted for 13.4% (11/82). These studies were classified as Level V literature because they were non-controlled studies, such as experience summaries and case reports. **Table 1** presents the basic characteristics of the literature.

**Table 1:** Literature basic characteristics.

Characteristic	Research type		Characteristic	Research type	
	Clinical experience (*n*=46)	Clinical observation (*n*=36)		Clinical experience (*n*=46)	Clinical observation(*n*=36)
Sample size			Identification of reference sources		
0-60	27	18	Guidelines	1	2
61-120	/	14	Textbook	/	4
121-180	/	3	94th edition of *Diagnostic Efficacy Criteria for Chinese Medicine Diseases*	/	11
>180	1	1	02nd edition of *Guiding Principles for Clinical Research of New Chinese Medicines*	/	4
NA	18	/	combined two and more	1	9
**The quality of the literature**			NA	44	6
Level I	/	25	**Types of Liver depression syndrome mentioned in the literature**		
Level II	/	11	1	42	35
Level III	/	/	More than one kind	4	1
Level IV	/	/			
Level V	46	/			

The bold text denotes the specific characteristics of the literature.

### Frequency of syndrome results

Analysis of 298 clinical entries revealed 25 distinct subtypes of Liver depression syndrome. Due to terminological variations across medical literature and practitioners, semantically equivalent subtypes were consolidated. The top five syndromes were: *qi* stagnation due to Liver depression (30.5%), phlegm congealing due to Liver depression (24.7%), Liver *qi* depression (19.7%), Liver depression and Spleen deficiency (15.6%) and stagnated Liver *qi* transforming into fire (3.7%). **Table 2** shows the main subtypes of Liver depression syndrome in breast cancer literature.

**Table 2:** Most prevalent subtypes of Liver depression syndrome in breast cancer literature.

TCM syndrome	Count	Percentage
Syndrome of *qi* stagnation due to Liver depression	90	30.5%
Syndrome of phlegm congealing due to Liver depression	73	24.7%
Syndrome of Liver *qi* depression	58	19.7%
Syndrome of Liver depression and Spleen deficiency	46	15.6%
Syndrome of stagnated Liver *qi* transforming into fire	11	3.7%
Syndrome of *yin* deficiency due to Liver depression	5	1.7%
Syndrome of Liver depression and Kidney deficiency	5	1.7%
Syndrome of blood stasis due to Liver depression	3	1.0%
Syndrome of stasis and toxin due to Liver depression	2	0.7%
Syndrome of Liver and Spleen stagnation	1	0.3%
Syndrome of toxin accumulation due to Liver depression	1	0.3%
Syndrome of stagnated *qi* transforming into heat due to Liver depression	1	0.3%
Syndrome of blood deficiency due to Liver depression	1	0.3%
Syndrome of motional internal injury	1	0.3%

### Frequency of signs and symptoms of liver depression syndrome in breast cancer

A total of 104 signs and symptoms associated with Liver depression syndrome in breast cancer were identified through systematic data curation. The top ten signs and symptoms were: distending pain in the chest and sub-costal region (68%), depression (67%), breast mass (64%), anger (52%), breast distending pain (44%), oppression in chest (44%), hard breast mass (34%), vexation (34%), bitter taste in mouth (33%), impatient (30%) (detailed in **Table 3**).

**Table 3:** Frequency of signs and symptoms of Liver depression syndrome evidence in breast cancer.

Symptoms	Frequency, n (%)	Symptoms	Frequency, n (%)
Distending pain in the chest and sub-costal region	203 (68)	Lack of strength	33 (11)
Depression	199 (67)	Symptoms vary depending on emotional states	25 (8)
Breast mass	190 (64)	Fixed breast lumps	23 (8)
Anger	156 (52)	Constipation	23 (8)
Breast distending pain	132 (44)	Menstrual disorders	19 (6)
Oppression in chest	130 (44)	Loose stool	17 (6)
Hard breast mass	102 (34)	Take less food	16 (5)
Vexation	101 (34)	Spontaneous sweating	16 (5)
Bitter taste in mouth	98 (33)	Lassitude	15 (5)
Impatient	90 (30)	Profuse dreaming	15 (5)
The skin color of the mass is unchanged	87 (29)	The surface of the mass is not smooth	14 (5)
Poor appetite	75 (25)	Breast lumps is not fixed	14 (5)
Dry throat	73 (24)	Tidal fever	14 (5)
Insomnia	62 (21)	Abdominal distension	13 (4)
The symptoms fluctuate depending on pre- and post-menstrual phases	60 (20)	Yellow, reddish urine	13 (4)
The boundary of the mass is unclear	57 (19)	Forgetfulness	13 (4)
Dizziness	57 (19)	Excessive thinking	12 (4)
Sighing	54 (18)	Migratory pain in the chest and sub-costal region	12 (4)
Dysphoria	43 (14)	Dysmenorrhea	12 (4)
Dry mouth	40 (13)	Speechless	11 (4)
Distending pain in the lower abdominal	39 (13)	Belching	11 (4)
Blurred vision	38 (13)	Nausea	10 (3)

In terms of tongue and pulse, this systematic review identified a total of 54 entries associated with Liver depression syndrome in breast cancer. The five most common tongue manifestations were: thin and white fur (48%), thin and yellow fur (28%), red tongue (23%), thin fur (17%), pale tongue (14%). The five most pulse conditions were: stringy pulse (41%), stringy and slippery pulse (28%), stringy and thready pulse (14%), stringy and deep pulse (7%), stringy and strong pulse (6%). **Table 4** presents the frequency distribution of tongue and pulse of Liver depression syndrome in breast cancer.

**Table 4:** The frequency of tongue manifestations and pulse conditions of Liver depression syndrome evidence in breast cancer.

Tongue manifestations	Frequency, n (%)	Pulse conditions	Frequency, n (%)
Thin, white fur	144 (48)	Stringy pulse	123 (41)
Thin, yellow fur	82 (28)	Stringy, slippery pulse	83 (28)
Red tongue	70 (23)	Stringy, thready pulse	42 (14)
Thin fur	52 (17)	Stringy, deep pulse	21 (7)
Pale tongue	43 (14)	Stringy, strong pulse	19 (6)
Pink tongue	29 (10)	Thready pulse	15 (5)
Dark red tongue	26 (9)	Stringy, rapid pulse	14 (5)
Yellow fur	26 (9)	Stringy, moderate pulse	9 (3)
Ecchymosis on tongue	25 (8)	Hesitant pulse	8 (3)
Dark tongue	15 (5)	Rapid pulse	7 (2)
White fur	13 (4)	Thready, rapid pulse	5 (2)
White, greasy tongue	12 (4)	Slippery pulse	5 (2)
Light yellow coating	11 (4)	Weak pulse	5 (2)
Dark purplish tongue	8 (3)	Thready, hesitant pulse	4 (1)
Teeth marks tongue	8 (3)	Deep, thready pulse	4 (1)
Greasy tongue	7 (2)	Stringy, hesitant pulse	3 (1)
Enlarged tongue	7 (2)	Stringy, thready and rapid pulse	3 (1)
Less fur	6 (2)	Moderate pulse	3 (1)
Pale white tongue	5 (2)	Deep, weak pulse	2 (1)
Thin, greasy coating	4 (1)		
Tender red tongue	3 (1)		
Purplish sublingual veins	3 (1)		
Peeling of tongue	2 (1)		

## Discussion

Traditional Chinese Medicine demonstrates therapeutic potential in breast cancer, with studies indicating its efficacy in alleviating side effects and improving quality of life (17–19). While Liver depression syndrome is a key clinical syndrome in breast cancer pathogenesis, the lack of standardized diagnostic criteria has hindered scientific understanding and TCM intervention development. This study systematically synthesizes literature to outline its clinical characteristics, dominant symptoms, and tongue–pulse diagnostic indicators, facilitating precise syndrome characterization and supporting TCM oncology theory refinement. Our study indicates that breast cancer patients with Liver depression syndrome exhibit six main symptom clusters: breast-related abnormalities, emotional disturbances, Liver meridian pathway manifestations, chest-abdominal discomfort, menstrual irregularities, and bowel/bladder dysfunction. Emotional symptoms, such as depression, anger, vexation, often fluctuating alongside emotional states.

The results of this study indicate that Liver depression syndrome in breast cancer presents with a variety of tongue and pulse manifestations. Across all literature related to Liver depression syndrome, thin white fur and stringy pulse are the most frequently observed. Tongue and pulse are critical diagnostic parameters in TCM syndromes. Previous studies have established several criteria for Liver depression syndrome across various diseases, yet tongue manifestations are seldom mentioned. For instance, tongue signs were not included in the diagnostic criteria for Liver depression and Liver *qi* depression syndromes (20, 21), suggesting that experts consider tongue signs to lack specificity for these patterns. In the criteria for Liver depression syndrome, the tongue description “pale tongue with thin white fur” represents normal physiological findings (22). This may reflect the TCM theory that tongue coating reflects Stomach *qi* transformation, while Liver depression primarily disrupts *qi* movement without directly inducing dampness or heat accumulation, leading to subtle tongue coating changes and reduced diagnostic value. In contrast, pulse conditions play a crucial role in the diagnosis of Liver depression syndrome. The stringy pulse is the only consistent indicator across criteria, aligning with our literature review findings.

TCM deeply understands Liver depression syndrome, identifying various causes such as external pathogens, emotional stress, and phlegm accumulation that disrupt the flow of vital energy *qi*. In breast cancer, Liver depression is both a common pattern and a crucial factor. TCM believes breast tissue is connected to the Liver meridian system, and breast health depends on the Liver and Stomach working together. Chronic emotional suppression or sudden anger can cause Liver *qi* stagnation, impairing Spleen function and nutrient processing. Combined with a diet high in rich or fatty foods, this can lead to internal phlegm-dampness buildup. When these harmful elements gather in breast tissue, they create the conditions for cancer to develop. Besides, emotional factors play a dominant role in Liver regulation, and Liver depression is linked to tumorigenesis in multiple ways. The Liver meridian traverses the throat, and prolonged Liver depression can generate fire-toxin. This fire-toxin scorches body fluids into phlegm, contributing to thyroid cancer (referred to as “gall tumors” in TCM) (23). Additionally, Liver depression and repressed anger induce *qi* stagnation and blood stasis, which are the pathological mechanisms underlying liver cancer (24).

In TCM, a classical view holds that Liver depression is a key pathogenic factor in breast cancer oncogenesis (25). However, since Liver depression syndrome comprises multiple subtypes, it remains unclear whether all subtypes contribute to breast cancer development. This uncertainty warrants further investigation. These subtypes reflect the progression of Liver depression through distinct pathological stages. TCM emphasizes “transformation of pathogenesis”, indicating that syndromes are not static but can evolve as the disease progress. Literature and textbooks (26, 27) show that in the early stage of breast cancer, Liver depression with *qi* stagnation is the main syndrome. Without timely intervention, it may gradually evolve into Liver depression transforming into fire, followed by Liver depression with blood stasis and phlegm coagulation. Prolonged Liver depression can damage Spleen function, leading to Spleen deficiency. Therefore, Liver depression with *qi* stagnation serves as the pathological foundation, while other syndrome manifestations are results of pathological evolution. Each subtype is not independently pathogenic but is jointly triggered by the pathological chain of “*qi* stagnation–phlegm stasis–toxic accumulation–vital deficiency”.

In terms of treatment, TCM focuses on getting the body’s energy *qi* flowing smoothly again by regulating the Liver. A prime example is the classic TCM formula Chaihu Shugan San, which relieves Liver congestion and improves emotional well-being and quality of life (28–30). With modern technological advancements, we can investigate specific effector pathways and molecular targets associated with Liver depression syndrome in breast cancer using histological and omics techniques (31). Our goal is to uncover the syndrome’s microscopic and molecular signatures. Additionally, using our team’s established Liver depression syndrome breast cancer mouse model, we can assess the efficacy of TCM interventions on key signaling nodes. This will provide a solid basis for precisely targeting Liver depression syndrome in breast cancer therapy.

Depressive symptoms in breast cancer patients are a significant public health issue (32–34), with a 30.2% prevalence globally in 2023 (35). There are significant clinical and pathophysiological links between TCM’s Liver depression syndrome and Western-defined depression in these patients. Modern biomedical research recognizes chronic stress as fundamentally analogous to TCM’s “Liver constraint” (Liver *qi* stagnation), as shown by preclinical studies using chronic stress models to induce Liver depression syndrome (36–38). Chronic psychological stress activates the sympathetic nervous system and the hypothalamic-pituitary-adrenal (HPA) axis, releasing substances that promote cancer progression (39) and impair immune surveillance (40), thereby increasing breast cancer susceptibility (41, 42). Additionally, experimental data show that chronic psychological stress accelerates mammary tumor growth and metastasis in mouse models, while the TCM formula *Sini San* counteracts these effects by reducing stress-induced aerobic glycolysis (43). The TCM pathomechanism of “*qi* stagnation” aligns with depression-associated dysregulation of the neuroendocrine-immune network. In TCM theory, the Liver regulates *qi* flow and emotions, and therapies that restore Liver *qi* dynamics likely exert multi-mechanistic effects: simultaneously normalizing *qi* circulation, alleviating emotional distress, and enhancing immune surveillance (44–46).

This study systematically maps the literature on Liver depression syndrome in breast cancer, but limitations exist. Most studies (56.1%) were Level V non-controlled, reflecting the current dominance of observational and case-based evidence in this field. Though valuable for clinical insights, their retrospective design and lack of control groups limit causal inference. Future research should focus on establishing standardized diagnostic criteria for Liver depression syndrome in breast cancer to enhance diagnostic accuracy and consistency. Utilizing advanced multi-omics technologies like metabolomics and spatial transcriptomics can help elucidate the syndrome’s biological mechanisms and identify key molecular events related to clinical depression. Integration of artificial intelligence (AI) and big data analytics will aid in developing objective biomarkers and early detection tools, advancing TCM syndrome standardization. Multicenter clinical trials are needed to validate the effects of Liver *qi*-regulating therapies on the tumor microenvironment and optimize herbal formulas. These efforts will promote the development of integrative TCM-Western treatment models to improve breast cancer prognosis.

## Data Availability

The original contributions presented in the study are included in the article/**Supplementary Material**. Further inquiries can be directed to the corresponding authors.
