# Correction: A systematic review and exploration of clinical application of Liver depression syndrome in breast cancer

**DOI:** 10.3389/fonc.2026.1820675

**Published:** 2026-03-12

**Authors:** Peng Wu, Siying Mao, Qian Zuo, Qianqian Guo, Qianjun Chen

**Affiliations:** 1 State Key Laboratory of Traditional Chinese Medicine Syndrome/Breast Disease Specialist Hospital, The Second Affiliated Hospital of Guangzhou University of Chinese Medicine, Guangdong Provincial Hospital of Chinese Medicine, Guangdong Provincial Academy of Chinese Medical Sciences, Guangzhou, Guangdong, China; 2 The Second Clinical College of Guangzhou University of Chinese Medicine, Guangzhou, Guangdong, China; 3 Guangdong Provincial Hospital of Chinese Medicine, The Second Affiliated Hospital of Guangzhou University of Chinese Medicine, Guangzhou, Guangdong, China; 4 Department of Breast, Guangdong Provincial Hospital of Chinese Medicine, Guangzhou, Guangdong, China; 5 Guangdong Provincial Key Laboratory of Clinical Research on Traditional Chinese Medicine Syndrome, Guangdong Provincial Hospital of Chinese Medicine, Guangzhou, Guangdong, China; 6 Postdoctoral Research Center, Guangdong Provincial Hospital of Chinese Medicine, Guangzhou, Guangdong, China; 7 Chinese Medicine Guangdong Laboratory, Guangzhou, Guangdong, China

**Keywords:** breast cancer, liver depression syndrome, systematic review, Chinese medicine syndrome, clinical application

There was a mistake in **Table 4** as published. In the fifth row from the bottom of the first column on the left of the table, the original text is “Pale tongue” but it should actually be “Pale white tongue” with one word missing, which is highlighted in below table in red. The corrected **Table 4** appears below.

**Table 4:** The frequency of tongue manifestations and pulse conditions of Liver depression syndrome evidence in breast cancer.

Tongue manifestations	Frequency, n (%)	Pulse conditions	Frequency, n (%)
Thin, white fur	144 (48)	Stringy pulse	123 (41)
Thin, yellow fur	82 (28)	Stringy, slippery pulse	83 (28)
Red tongue	70 (23)	Stringy, thready pulse	42 (14)
Thin fur	52 (17)	Stringy, deep pulse	21 (7)
Pale tongue	43 (14)	Stringy, strong pulse	19 (6)
Pink tongue	29 (10)	Thready pulse	15 (5)
Dark red tongue	26 (9)	Stringy, rapid pulse	14 (5)
Yellow fur	26 (9)	Stringy, moderate pulse	9 (3)
Ecchymosis on tongue	25 (8)	Hesitant pulse	8 (3)
Dark tongue	15 (5)	Rapid pulse	7 (2)
White fur	13 (4)	Thready, rapid pulse	5 (2)
White, greasy tongue	12 (4)	Slippery pulse	5 (2)
Light yellow coating	11 (4)	Weak pulse	5 (2)
Dark purplish tongue	8 (3)	Thready, hesitant pulse	4 (1)
Teeth marks tongue	8 (3)	Deep, thready pulse	4 (1)
Greasy tongue	7 (2)	Stringy, hesitant pulse	3 (1)
Enlarged tongue	7 (2)	Stringy, thready and rapid pulse	3 (1)
Less fur	6 (2)	Moderate pulse	3 (1)
**Pale white tongue**	5 (2)	Deep, weak pulse	2 (1)
Thin, greasy coating	4 (1)		
Tender red tongue	3 (1)		
Purplish sublingual veins	3 (1)		
Peeling of tongue	2 (1)		

The original version of this article has been updated.

