# Supplementary material for: A systematic review and exploration of clinical application of liver depression syndrome in breast cancer
Source: Front Oncol. 2025 Aug 11;15:1614903. doi: 10.3389/fonc.2025.1614903 (PMC12375494; doi:10.3389/fonc.2025.1614903)
Supplement: Supplementary file 1 [file DataSheet1.docx]

**Supplementary material 1**

Table S1 Search terms for published research articles

| Database | Search terms |
| --- | --- |
| Chinese search terms | SU %= ( '乳腺癌' + '乳岩' + '乳癌' ) AND SU %=( '肝郁' + '肝郁气滞' + '肝气郁结' + '肝郁气逆' + '肝郁痰凝' + '肝郁化热' + '肝郁化火' + '肝郁火旺' + '肝郁痰热' + '肝郁痰火' + '肝郁湿热' + '肝郁血热' + '肝郁血瘀' + '肝郁血虚' + '肝郁阴虚' + '肝郁脾虚' + '肝郁肾虚' + '肝胆郁热' ) |
| English search terms | Contains the following keywords: “breast neoplasms”, “breast cancer”, “breast carcinoma”, “breast tumor”, “stagnation of the Liver”, “syndrome of Liver *qi* depression”, “syndrome of Liver depression with *qi* counterflow”, “syndrome of *qi* stagnation due to Liver depression”, “syndrome of stagnated *qi* transforming into heat due to Liver depression”, “syndrome of stagnated Liver *qi* transforming into fire”, “syndrome of Liver stagnation and fire blazing”, “syndrome of phlegm congealing due to Liver depression”, “syndrome of phlegm-heat due to Liver depression”, “syndrome of phlegm-fire due to Liver depression”, “syndrome of dampness and heat due to Liver depression”, “syndrome of blood heat due to Liver depression”, “syndrome of blood stasis due to Liver depression”, “syndrome of blood deficiency due to Liver depression”, “syndrome of *yin* deficiency due to Liver depression”, “syndrome of Liver depression and Spleen deficiency”, “syndrome of Liver depression and Kidney deficiency”, “syndrome of stagnated heat in the Liver and Gallbladder” |
