# Supplementary figures and images for: A systematic review and exploration of clinical application of liver depression syndrome in breast cancer
Source: Front Oncol. 2025 Aug 11;15:1614903. doi: 10.3389/fonc.2025.1614903 (PMC12375494; doi:10.3389/fonc.2025.1614903)

**Supplementary material 2**

Figure 1. Graphical abstract of the study


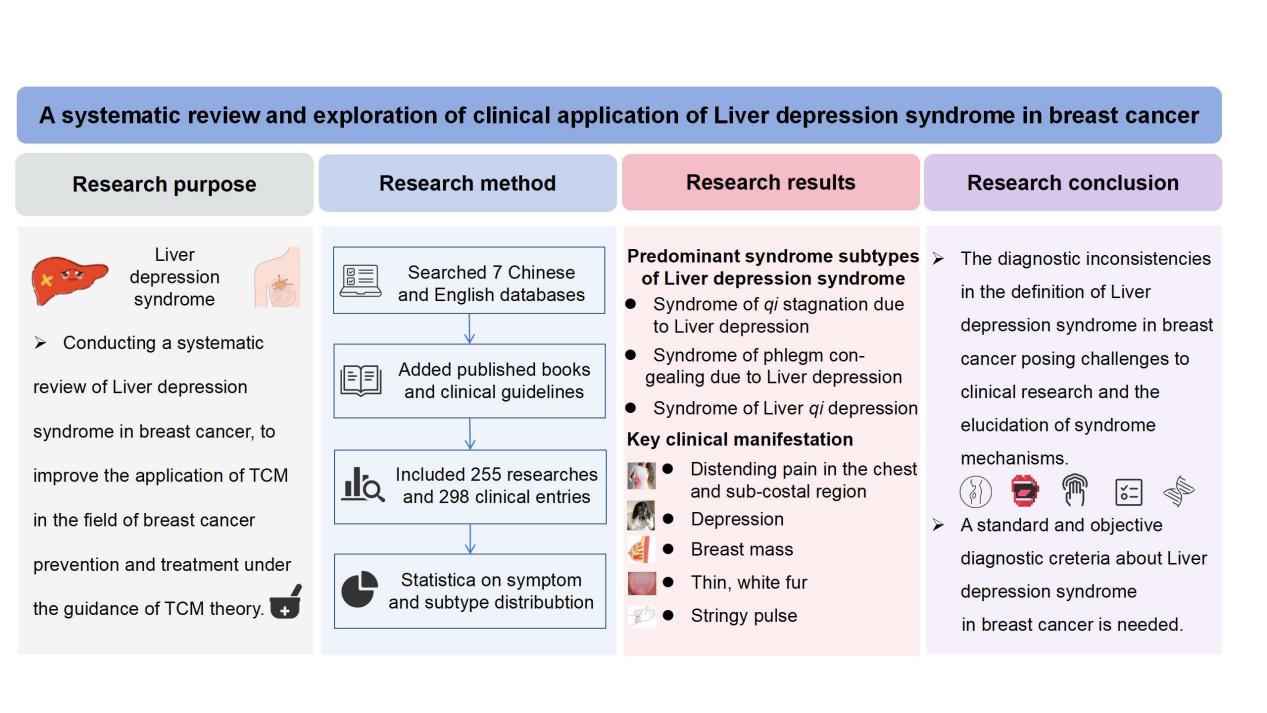

Supplement: Supplementary file 2 [file DataSheet2.docx]
